# Combination of STING and TLR 7/8 Agonists as Vaccine Adjuvants for Cancer Immunotherapy

**DOI:** 10.3390/cancers14246091

**Published:** 2022-12-11

**Authors:** Shubhmita Bhatnagar, Vishnu Revuri, Manan Shah, Peter Larson, Zekun Shao, Daohai Yu, Swayam Prabha, Thomas S. Griffith, David Ferguson, Jayanth Panyam

**Affiliations:** 1 Department of Pharmaceutical Sciences, School of Pharmacy, Temple University, Philadelphia, PA 19140, USA; 2 Department of Pharmaceutics, College of Pharmacy, University of Minnesota, Minneapolis, MN 55455, USA; 3 Department of Medicinal Chemistry, College of Pharmacy, University of Minnesota, Minneapolis, MN 55455, USA; 4 Center for Biostatistics and Epidemiology, Lewis-Katz School of Medicine, Temple University, Philadelphia, PA 19140, USA; 5 Fels Cancer Institute for Personalized Medicine, Lewis-Katz School of Medicine, Temple University, Philadelphia, PA 19140, USA; 6 Fox Chase Comprehensive Cancer Institute, Temple University, Philadelphia, PA 19111, USA; 7 Department of Urology, Medical School, University of Minnesota, Minneapolis, MN 55455, USA

**Keywords:** cancer immunotherapy, cancer vaccine, toll-like receptors, STING, DMXAA

## Abstract

**Simple Summary:**

The clinical use of immunoadjuvants is limited by transient responses and various side effects. This study investigated the multi-adjuvant approach of combining a TLR 7/8 agonist (522) and a STING agonist (DMXAA) to generate a robust anticancer immune response. Immunization with ovalbumin+DMXAA+522 resulted in the activation of DCs in lymph nodes, spleen, and tumor. The combination also elicited stronger antigen-specific CD8+ T cell and NK cell responses than the control or individual treatments. Immunization with OVA+DMXAA+522 resulted in significant tumor growth inhibition and improved survival compared to other controls.

**Abstract:**

Immunostimulatory adjuvants that potently activate antigen-presenting cells and (in turn) prime cytotoxic T cells are a key component of anticancer vaccines. In this study, we investigated a multi-adjuvant approach combining a TLR 7/8 agonist (522) and a STING agonist (DMXAA) to promote enhanced antigen cross-presentation, stimulate specific antitumor T-cell responses, and provide improved anticancer efficacy. In vitro experiments using bone marrow-derived dendritic cells (BMDCs) confirmed enhanced activation with the 522-DMXAA combination based on both co-stimulatory molecule expression and pro-inflammatory cytokine secretion. The immunization of mice with vaccines comprising both 522 and DMXAA resulted in greater antitumor efficacy in B16F10 melanoma and MB49 bladder tumor models relative to mono-agonist vaccines. Flow cytometry-based analysis of immune cells from immunized mice revealed the significant activation of antigen-presenting cells, increased numbers of activated and Ag-specific CD8+ T cells in the spleen and lymph nodes, modest NK cell activation, and an overall reduction in CD206^+^ macrophages. These results were supported by an increase in the levels of IFN-γ and a reduction in IL-10 levels in the sera. Taken together, these findings demonstrate the potential of the TLR7/8 and STING agonist combination as vaccine adjuvants to activate both innate and adaptive immune responses.

## 1. Introduction

The activation of antigen (Ag)-specific antitumor immune responses is critically dependent upon the context in which a tumor antigen is presented by antigen-presenting cells (APCs). Antigen presentation must be accompanied by ‘danger’ signals to properly activate cytotoxic T cells. Such ‘danger’ signals are provided primarily by pathogen recognition receptors (PRRs) expressed by APCs [1,2]. The activation of PRRs results in the expression of chemokines, co-stimulatory ligands, and cytokines, which together form the ‘danger’ signal and result in the effective priming of antitumor CD8+ T cells [3,4].

While several PRR families have been identified, toll-like receptors (TLR) are the most extensively studied [5]. Among the 10 identified human TLR sub-types, TLR7/8 are particularly interesting because they can be activated by small molecules in addition to single-stranded RNA. Imiquimod, a TLR7-specific small molecule agonist, is approved for the topical treatment of basal cell carcinoma. Several other TLR agonists are in clinical trials [6,7,8]. More recently, there has been significant interest in developing agonists that can activate cyclic the GMP–AMP synthase (cGAS)-Stimulator of IFN Genes (STING) pathway, a cytosolic PRR. Many STING agonists are in preclinical and clinical development [9].

**Figure 1:**
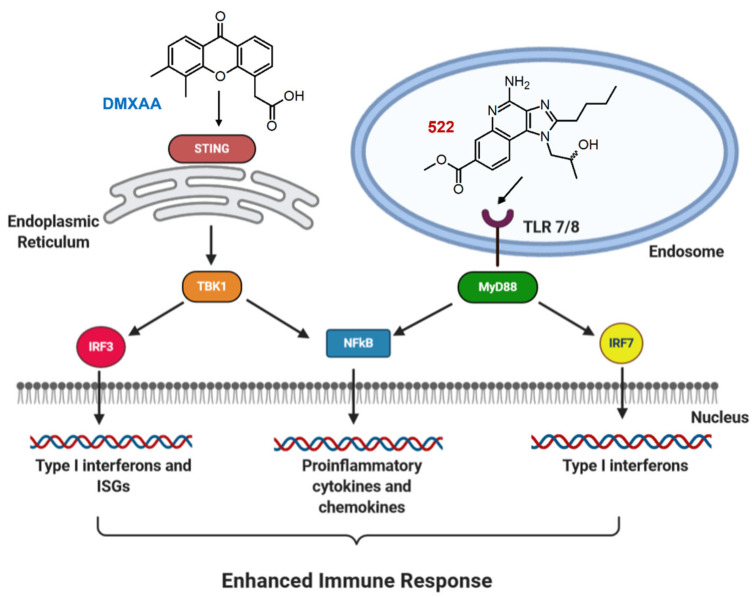
Proposed combination adjuvant therapy for cancer vaccination. Briefly, 522 is a TLR7/8 agonist that is more potent than imiquimod in activating APCs. DMXAA is a mouse STING-specific agonist that has previously been shown to activate type I interferon response in APCs.

Following agonist binding, TLRs activate myeloid differentiation primary-response gene 88 (MyD88) along with toll/interleukin-1 receptor (TIR) domain-containing adaptor protein inducing Interferon-β (IFN- β) (TRIF) signaling pathways [5]. The downstream signaling leads to the nuclear translocation of the NF-κB and IRF3/7 transcription factors, and the regulation of genes encoding pro-inflammatory cytokines and type-I interferons (Figure 1) [10]. On the other hand, STING agonists stimulate the activation of TANK-binding kinase 1 (TBK1) and IRF3, inducing the transcription of genes encoding type I interferons [11]. The activation of the STING pathway also results in the activation of NF-κB and the transcription of proinflammatory cytokines such as interleukin-6 (IL-6), tumor necrosis factor (TNF), and IFN-α [12,13].

We hypothesized that the concurrent stimulation of the TLR7/8 and STING pathways would result in the improved activation of APCs, enhanced CD8+ T cell priming, and greater anticancer efficacy. To test this hypothesis, we used the novel imidazoquinoline-based TLR7/8 agonist 522 and DMXAA, a mouse-specific STING agonist [14,15,16,17,18]. Using in vitro bone marrow-derived dendritic cell (BMDC) assays and in vivo tumor models, we examined the effectiveness of the DMXAA-522 combination in activating APCs and eliciting an anti-tumor immune response. Our studies show DMXAA-522 is more effective than individual agonists in activating the expression of pro-inflammatory cytokines and co-stimulatory molecules, resulting in greater CD8+ T cell priming and anti-tumor activity.

## 2. Experimental

### 2.1. Materials

The TLR 7/8 agonist 522 was synthesized and characterized as reported previously [15,19]. DMXAA was purchased from Cayman Chemicals (Ann Arbor, MI, USA). Fluorophore-labeled monoclonal antibodies (CD8, CD49b, NKG2D, CD3, CD45, CD69, CD44, CD4, F4/80, CD206, CD80, H-2Kb, CD40, CD11c, CD86, CD19, I-A/I-E(MHC II)) and fluorophore-labeled OVA_257–264_ (SIINFEKL) H-2K^b^ tetramer (Item 280051) were purchased from Biolegend (San Diego, CA, USA). SIINFEKL peptide (AS-60193-1) was purchased from Anaspec Inc. (Fremont, CA, USA).

### 2.2. Animals and Cell Line

All animal experiments were performed according to the protocols approved by the Institutional Animal Care and Use Committee (IACUC) of Temple University.

C57BL/6J mice (6–8 weeks, female) were purchased from Charles River (Wilmington, MA, USA) and housed under specific pathogen-free facilities maintained by the University Laboratory and Animal Resources at Temple University. Ovalbumin (OVA) expressing murine melanoma cell line B16F10-OVA was provided by Dr. Brandon Burbach (University of Minnesota). Mouse bladder cancer cell line MB49 was procured from ATCC. B16F10-OVA and MB49 cells were maintained in complete RPMI 1640 medium (RPMI 1640 with 10% fetal bovine serum, 100 µg/mL streptomycin, and 100 U/mL penicillin). Medium was supplemented with G-418 Disulfate (Research products international, Mt Prospect, IL, USA) when culturing B16F10-OVA (250 µg/mL).

### 2.3. Culture of BMDCs

Tibias and femurs from C57BL/6J mice were harvested, disinfected with 70% ethanol, and rinsed twice with cold PBS (pH 7.4). After snipping both ends of the bone, the bone marrow was flushed with PBS using a 27-gauge needle (Medtronic, Minneapolis, MN, USA), passed through a 70-µm cell strainer, and red blood cells were removed using lysis buffer (Gibco, Waltham, MA, USA). To generate immature BMDCs, single cell suspension of bone marrow precursor cells was incubated with complete RPMI 1640 media supplemented with 20 ng/mL granulocyte-macrophage colony-stimulating factor (GM-CSF) (PeproTech, Rocky Hill, NJ, USA) and 50 μM 2-mercaptoethanol (Sigma) for 6 days. Media was changed on day 3. BMDCs were characterized as CD11c^+^ MHC II^+^ cells.

### 2.4. In Vitro BMDC Activation

BMDCs (10^6^/well/mL) were seeded in a 24-well cell culture plate overnight, followed by incubation with different treatments. After 24 h, supernatants were analyzed for cytokine (TNF-α, IL-6, IL-10, IL-12) secretion using ELISA (Biolegend, San Diego, CA, USA), and BMDCs were analyzed for co-stimulatory molecule (CD40, CD86, and CD80) expression by flow cytometry [20].

### 2.5. Immunization Protocol

**Figure 2:**
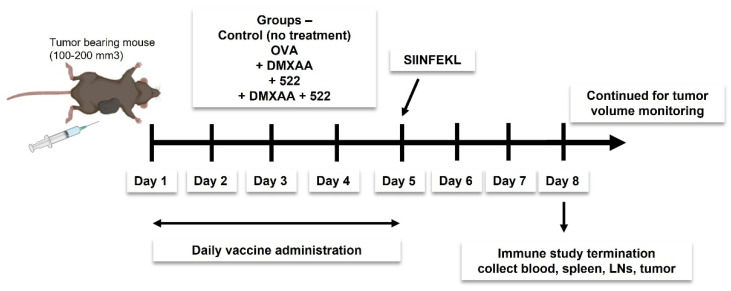
Immunization protocol.

We investigated two different vaccine strategies. The first approach utilized OVA as a model antigen, while the second strategy utilized tumor cell lysate as the source of tumor antigens. Mice were immunized daily for 5 days (Figure 2) using a mixture of OVA (100 µg) and 522 (5 µg), with or without DMXAA (50 µg) dispersed in 100 µL of sterile PBS. Doses were divided into two 50 µL subcutaneous injections and administered to the left and right thigh. The study was either terminated on day 8 to collect various tissues (blood, spleen, draining lymph nodes, and tumor) for immune studies or continued until the endpoint (tumor necrosis, death, or tumor size ≥ 2000 mm^3^) to investigate antitumor efficacy. The immunization protocol was based on previous studies that showed a minimum of five doses administered once a day was optimal for eliciting an effective antitumor immune response [14,21].

For preparing tumor cell lysate-based vaccine formulation, MB49 cells were subjected to five freeze–thaw cycles. Cell debris was centrifuged, and the protein concentration of the supernatants was measured using a BCA Assay Kit (Pierce™, ThermoScientific). The pellets were reconstituted with supernatants. Lysate (equivalent to 100 µg cell protein) was mixed with 522 (5 µg) and/or DMXAA (50 µg) in 100 µL of sterile PBS to create the vaccine. Vaccination dosing regimen and injection route were similar to those used for OVA immunization.

### 2.6. In Vivo Antitumor Efficacy

Mice were injected subcutaneously near the right thigh with B16F10-OVA cells (2 × 10^5^ cells/mouse) or MB49 cells (2 × 10^5^ cells/mouse) suspended in 100 µL PBS. Once the tumors reached a volume ≥100 mm^3^, the mice were immunized as described before. Tumor dimensions were determined every 2–3 days using a Vernier caliper. The tumor volume was calculated as V = 0.5 × (l × b^2^) (l: longest diameter, b: shortest diameter). Mice with tumor volumes >2000 mm^3^ or those that developed tumor ulceration were removed from the study and euthanized. Ulceration and euthanasia due to worsening disease conditions were considered death events for statistical purposes.

### 2.7. In Vivo APC Activation and T Cell Proliferation Assay

Mice bearing B16F10-OVA tumors (150–200 mm^3^) were immunized as described above for 5 days. On day 5, mice also received 100 µg of OVA_257–264_ peptides subcutaneously. On day 8, mice were sacrificed, and tissues were collected. Serum was separated from the blood and stored at −80°C until further use. Single-cell suspensions were prepared from the spleen, lymph nodes, and tumor. A portion of the tumor tissue was fixed using 10% formalin and processed for immunohistochemistry. The rest of the tumor was minced into smaller pieces using a sharp blade and digested in 2–3 mL RPMI media containing hyaluronidase (300 U/mL) and collagenase (250 U/mL) using a gentleMACS^TM^ Dissociator. The sample was passed through a cell strainer and treated with DNase (200 U/mL) followed by red blood cell lysis. The cell suspensions were then stained with anti-CD8, -CD49b, -NKG2D, -CD3, -CD45, -CD69, -CD44, -CD4, -F4/80, -CD206, -CD80, -CD40, -CD11c, -CD86,and -CD19 antibodies as well as OVA_257–264_: H-2K ^b^ tetramer-APC. The complete list of markers used to identify different cell populations and gating strategies is provided in Supplementary Figure S1. Serum cytokine levels were measured using a 44-plex Multiplex Assay (Eve Technologies Corporation, Calgary, AB, Canada).

### 2.8. Immunohistochemistry

Tumors were fixed using 10% formalin for 24–48 h and stored in 70% ethanol until embedding into paraffin blocks and sectioning. Tumor sections were stained with an anti-caspase-3 antibody.

### 2.9. Statistical Analyses

The results are presented as either mean ± standard deviation (SD) or mean ± standard error of the mean (SEM). Mixed-effects regression model or repeated measures analysis of variance (ANOVA) with post hoc Tukey’s test was used to determine the statistical significance of the observed differences between the treatment groups unless otherwise noted. Tumor growth (slopes) was compared between treatment groups using a mixed-effects model with Bonferroni correction in SAS. For survival analyses, groups were compared using a log-rank test with survival probability at 15 days and its confidence interval reported. A *p*-value ≤0.05 was considered statistically significant; *p*-values were indicated using the following scheme: * *p* ≤ 0.05, ** *p* ≤ 0.01, *** *p* ≤ 0.001, **** *p* ≤ 0.0001, n.s = not significant (*p* > 0.05). Data were analyzed using GraphPad Prism 8 and SAS version 9.4 software.

## 3. Results

### 3.1. DMXAA and 522 Combination Enhances BMDC Activation

**Figure 3:**
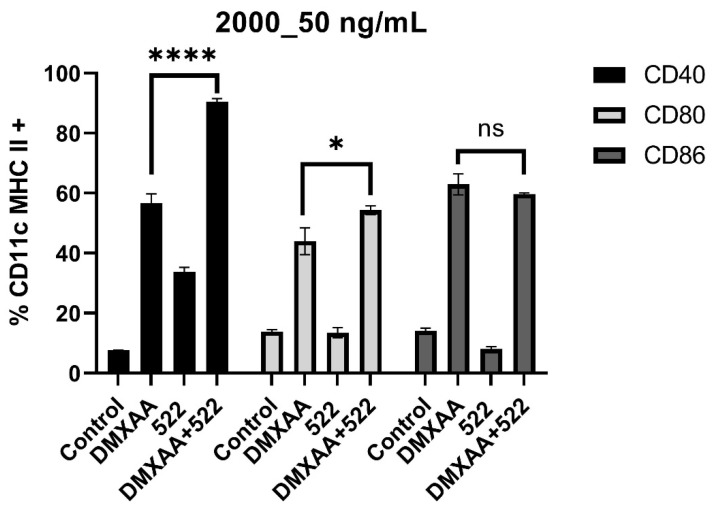
BMDC costimulatory molecule expression and cytokine secretion. BMDCs were incubated with DMXAA (2000 ng/mL), 522 (50 ng/mL), and DMXAA + 522 (2000_50) for 24 h, and costimulatory molecules CD40, CD80, and CD86 were measured by flow cytometry. CD11c and MHCII double-positive cells were identified as BMDCs in the population and were gated further for analysis. Results are reported as mean ± SD, *n* = 3, * *p* < 0.05, **** *p* < 0.0001, ns = not significant (*p* > 0.05).

We initially examined whether the combination of DMXAA and 522 could result in the enhanced activation of BMDCs by determining the effect of the combination on costimulatory molecule expression and proinflammatory cytokine secretion. BMDCs were treated with varying concentrations of DMXAA (1000 and 2000 ng/mL), 522 (50, 100, and 200 ng/mL), and combinations of DMXAA and 522 (1000_100 ng/mL, 1000_50 ng/mL, 2000_50 ng/mL, 2000_200 ng/mL). Combination treatment led to a significant increase in CD40 expression and a small upregulation of CD80 and CD86 as compared to that with individual agonists (Figure 3 and Supplementary Figure S2).

**Figure 4:**
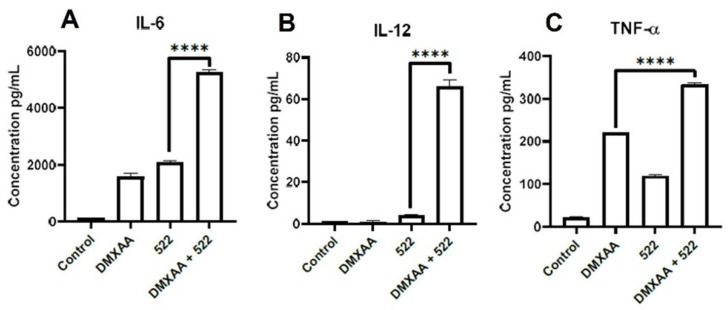
Cytokine secretion. After incubation of BMDCs with DMXAA (2000 ng/mL), 522 (50 ng/mL), and DMXAA + 522 (2000_50) for 24 h, cell culture supernatants were analyzed for IL-6 (**A**), IL-12 (**B**), and TNF-α (**C**) using ELISA. Results are reported as mean ± SD, *n* = 3, **** *p* < 0.0001.

A key characteristic of TLR7/8 and STING activation is the induction of pro-inflammatory cytokine expression. The treatment of BMDCs with DMXAA (2000 ng/mL), 522 (50 ng/mL), or the DMXAA + 522 combination (equivalent concentrations) resulted in significantly enhanced secretions of TNF-α, IL-6, and IL-12 (Figure 4A–C). The cytokine levels (especially IL-6 and IL-12) following treatment with the combination were more than the sum of the cytokine levels obtained with the individual treatments, suggesting that the effects were synergistic. The levels of IL-10 were, however, also observed to be enhanced with combination treatments of BMDCs (Supplementary Figure S2D).

### 3.2. Therapeutic Vaccination with DMXAA + 522 Combination Enhances Tumor Inhibition and Improves Survival

**Figure 5:**
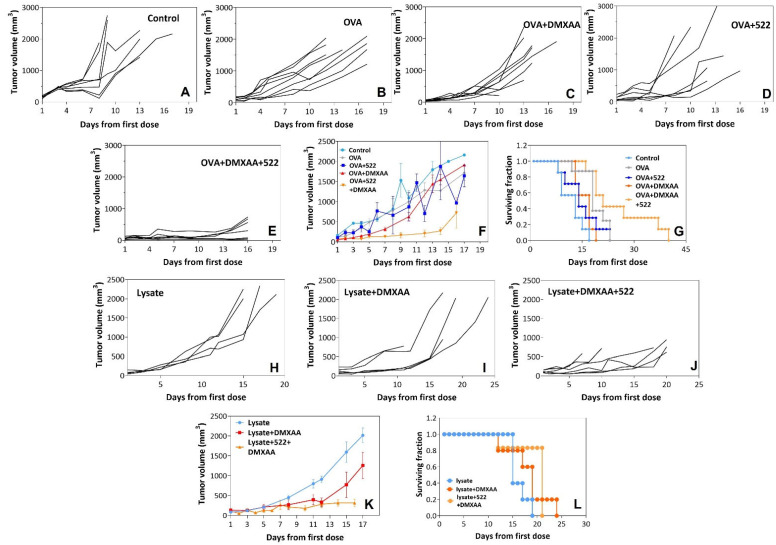
Anti-tumor efficacy using B16F10-OVA and MB49 tumor models. Mice bearing B16F10-OVA tumors were injected subcutaneously with five doses of OVA alone or OVA along with 522, DMXAA, or DMXAA + 522 (**A**–**G**). Mice bearing MB49 tumors were injected subcutaneously with five doses of cell lysate, DMXAA + lysate, or DMXAA + 522 + cell lysate (**H**–**L**). Data presented as tumor volumes vs. time since treatment for each mouse (**A**–**E**; **H**–**J**). Data presented as mean ± SEM for different treatment groups with B16F10-OVA (**F**) and MB49 (**K**) tumors. Kaplan–Meier survival curves for the different treatment groups with B16F10-OVA (**G**) and MB49 tumors (**L**).

The therapeutic effectiveness of the DMXAA + 522/OVA vaccine was examined in the B16F10-OVA tumor model. Tumors in both untreated and OVA-treated mice grew quickly; all control mice had tumors ≥1000 mm^3^ by d 10 (Figure 5A). Immunization with DMXAA + 522/OVA resulted in significant (*p* < 0.001) tumor growth inhibition compared to that with any of the other treatment groups and control. At d 12, the average tumor volume of the OVA-treated group and the DMXAA + 522/OVA groups were 1288 and 129 mm^3^, respectively. Treatment with DMXAA/OVA also resulted in significant tumor growth inhibition (*p* < 0.001) compared to the untreated control; however, the therapeutic benefit was less than that compared to the DMXAA + 522/OVA treatment. Correlating with enhanced tumor growth inhibition, immunization with the DMXAA + 522 adjuvant combination significantly enhanced survival compared to other treatments (vs. DMXAA+: *p* < 0.001; vs. 522+: *p* = 0.01; survival comparison using a log-rank test; Figure 5F), further demonstrating the anti-tumor efficacy of the TLR7/8 and STING agonist combination.

The effectiveness of the DMXAA + 522 combination to prime antitumor immunity against MB49 murine bladder tumors using whole tumor cell lysate as a relevant antigen source was evaluated next. This study also demonstrated the superior (*p* < 0.001) antitumor effectiveness of tumor growth inhibition for the combination vaccines (cell lysate + DMXAA + 522) compared to the DMXAA- or lysate-based vaccine (Figure 5G–I). The combination treatment resulted in a slight improvement in median survival (vs. lysate: *p* < 0.05) (Figure 5J). However, no statistically significant survival advantage was identified between the lysate + DMXAA + 522 and lysate + DMXAA treatment groups (*p* = 0.11) or between the lysate + DMXAA and lysate-only groups (*p* = 0.12).

### 3.3. Immunohistology

**Figure 6:**
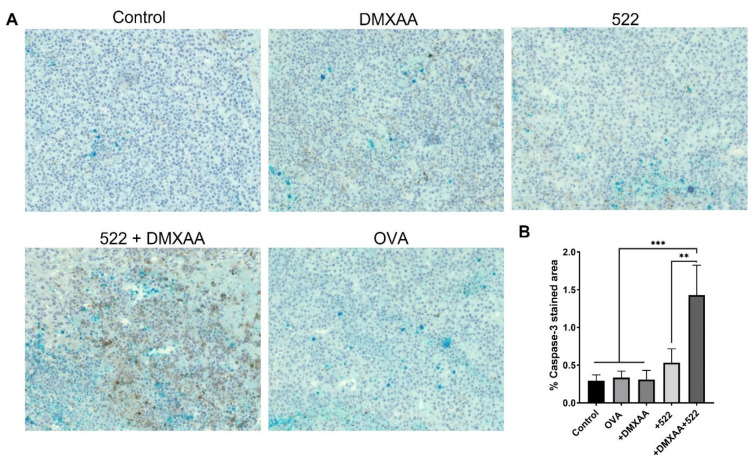
Induction of apoptosis with 522+ DMXAA combination vaccine. Anti-caspase-3 mAb staining of tumor sections collected on day 8 to detect apoptosis (**A**). The apoptotic cells are stained cyan (red arrow). Images were acquired at 10× magnification. These panels are representative of the sections that were analyzed for each cohort. The cyan stain areas were quantified using ImageJ (**B**). ** *p* < 0.01, *** *p* < 0.001, one-way ANOVA. *n* = 3.

Tumors excised on day 8 after treatments were stained with an anti-caspase-3 mAb to detect the induction of apoptosis. Cyan-stained areas quantified using ImageJ demonstrated higher percentages of caspase-3-stained areas in DMXAA + 522/OVA immunization compared to individual treatments (Figure 6), correlating with the improved anticancer efficacy of the combination treatment.

### 3.4. Activation of APCs In Vivo

**Figure 7:**
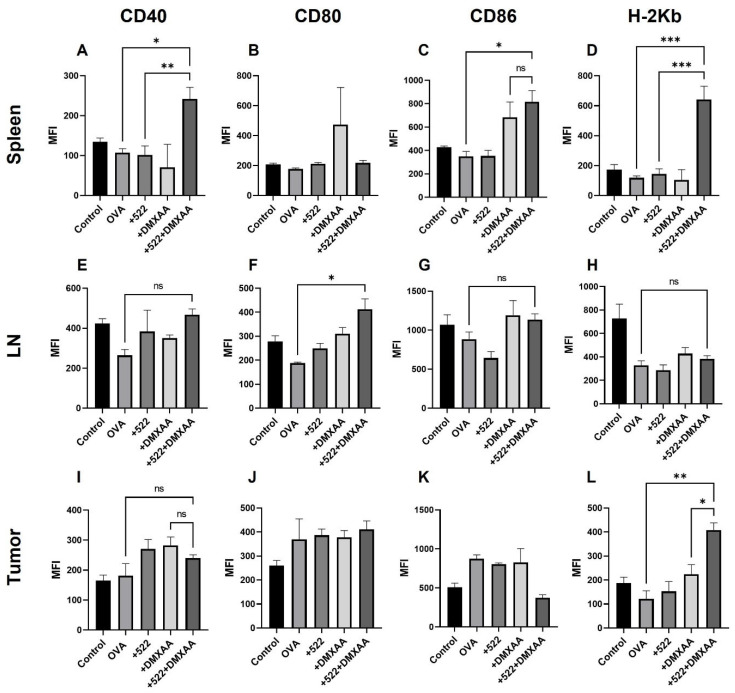
Activation of DCs in vivo. DCs in the spleen (**A**–**D**), lymph nodes (**E**–**H**) and tumor (**I**–**L**) were analyzed for the co-stimulatory molecule expression and MHC I presentation of OVA-derived SIINFEKL peptide (H-2K ^b^) by flow cytometry. Data presented as mean ± SD, *n* = 2–4. One-way ANOVA * *p* < 0.05, ** *p* < 0.01, *** *p* < 0.001, ns = not significant.

The rapid expansion and activation of DCs in lymph nodes are critical for the initiation of the adaptive immune response [22]. DC activation was confirmed by monitoring costimulatory molecule expression (CD80, CD86, and CD40) using flow cytometry. Interestingly, the different treatments affected the three costimulatory molecules differently. Treatment with DMXAA + 522 resulted in the increased expression of both CD40 and CD86 on DCs isolated from the spleen while having no effect on CD80 expression (Figure 7A–C). Combination treatment also significantly increased the MHC I presentation of the OVA-derived SIINFEKL peptide (Figure 7D). For DCs isolated from the draining lymph node, none of the treatments had any effect on the CD40 or CD80 expression compared to the untreated control, while treatment with the combination resulted in a significant increase in CD86 expression (Figure 7E–G). None of the treatments had any effect on OVA presentation by DCs in the lymph node (Figure 6H). None of the treatments had any effect on CD40, CD80, or CD86 expression on DCs isolated from the tumor (Figure 7I–K) while the combination treatment significantly increased the MHC I antigen presentation on these cells (Figure 7L).

**Figure 8:**
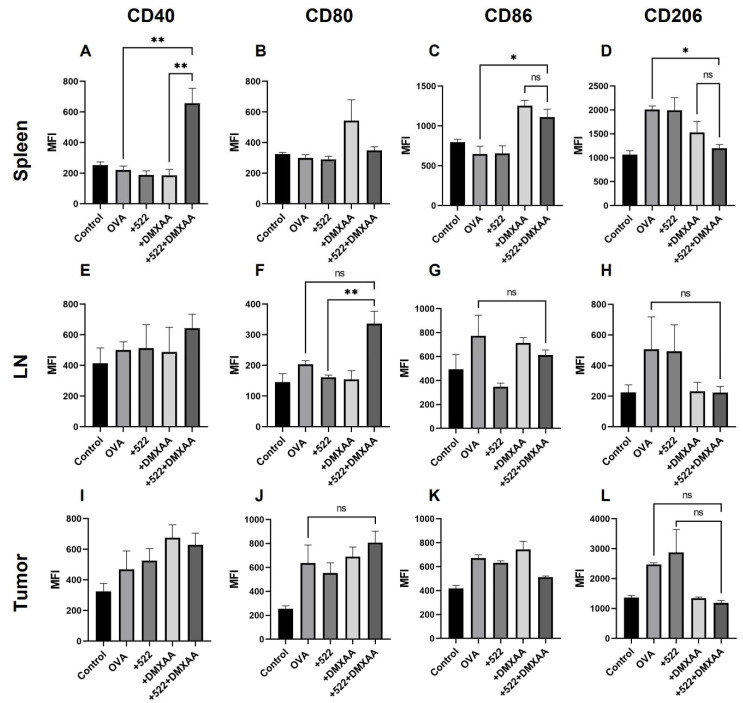
Activation of macrophages in vivo. Macrophages in the spleen (**A**–**D**), lymph nodes (**E**–**H**), and tumor (**I**–**L**) were analyzed for the expression of CD40, CD80, CD86 and CD206. Data presented as mean ± SD, *n* = 2–4. One-way ANOVA * *p* < 0.05, ** *p* < 0.01, ns = not significant.

Similarly, the tissue-dependent activation of macrophages was observed with DMXAA + 522 treatment (Figure 8). For example, the combination treatment resulted in CD40 and CD86 activation in splenic macrophages (Figure 8A–C) and CD80 activation in lymph node-derived macrophages compared to individual agonists (Figure 8E–G). All the treatments including OVA resulted in expressions of CD40 and CD80 but not CD86 in tumor-derived macrophages (Figure 8I–K). However, we found a consistent reduction in the number of CD206^+^ M2 macrophages after immunization with DMXAA + 522. In the spleen, CD206 expression on macrophages was significantly reduced with DMXAA + 522 compared to that with the OVA-only-treated group (Figure 8D). CD206^+^ macrophages were also reduced in the lymph nodes and tumor cells; however, the differences were not statistically significant (Figure 8H,L).

**Figure 9:**
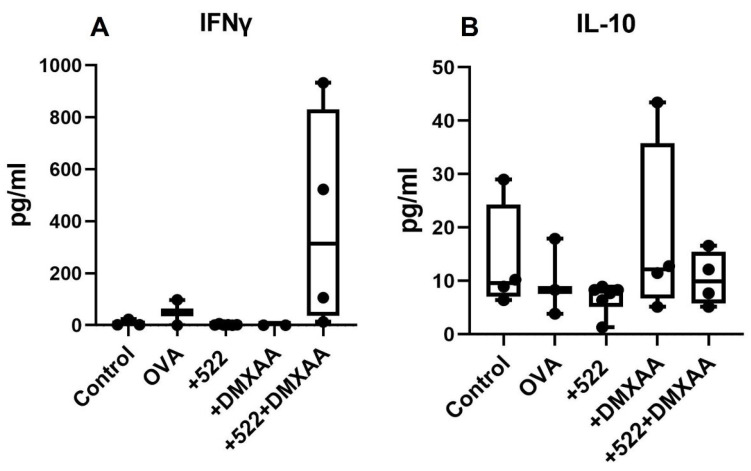
Serum cytokine levels. Levels of IFNγ (**A**) and IL-10 (**B**) in the serum on day 7 after 5 consecutive doses of different treatments on day 1 through day 5 were measured using Luminex.

We also evaluated the effect of the above treatments on systemic cytokine induction. Mice treated with DMXAA + 522 had increased levels of IFN-γ (Figure 9A), a key Th1 cytokine that stimulates T cell activation, and is crucial for the generation of anti-tumorigenic immune responses. In contrast, the IL-10 levels were comparatively lower in the combination group (Figure 9B). The levels of various cytokines measured are shown in Supplementary Figure S3.

### 3.5. Antigen-Specific CD8+ T Cell Activation

**Figure 10:**
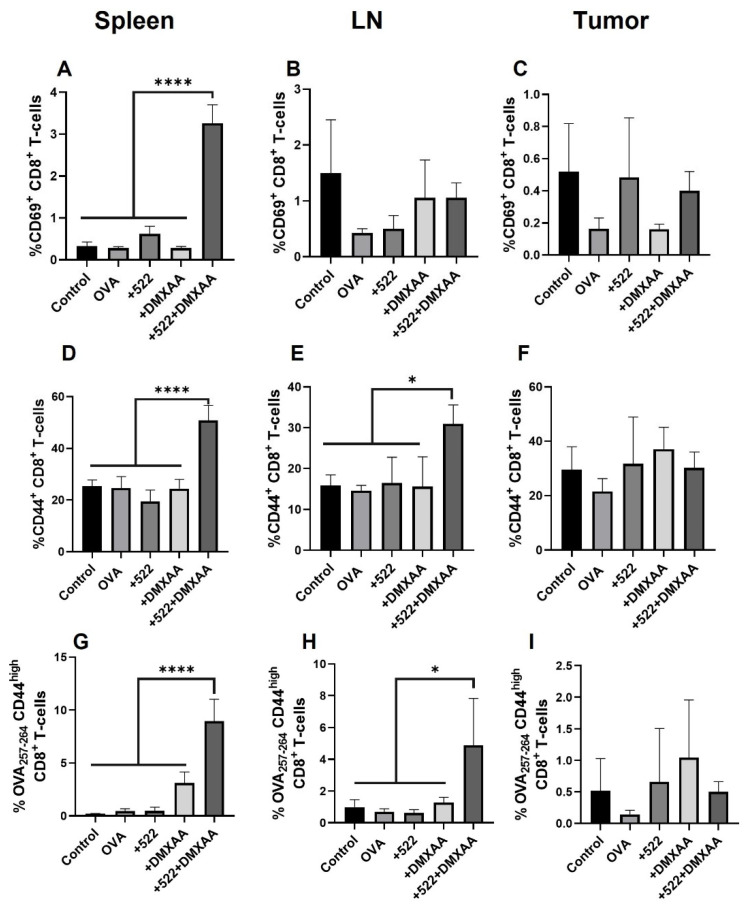
Activation of T-cells. T-cells from the spleen (**A**,**D**,**G**), lymph nodes (**B**,**E**,**H**) and tumor (**C**,**F**,**I**) were analyzed. OVA/DMXAA + 522 immunization significantly increased the number of OVA-specific CD44^high^ CD8+ T cells to other treatment groups and control mice in the spleen and lymph nodes but not in the tumor. **** *p* < 0.0001, * *p* < 0.05. One-way ANOVA, *n* = 3–4. LN: lymph nodes.

In addition to determining the overall T cell activation, we examined the DMXAA + 522-induced priming of OVA-specific CD8+ T cells using OVA_257–264_:H-2K^b^ tetramer [23]. Antigen-specific CD8+ T cells were also gated for the early activation marker CD69 and memory/effector marker CD44. We observed an increase in both activated CD69^+^ CD8+ T cells as well as CD44^high^ CD8+ T cells in the spleen and draining lymph nodes but not in tumors (Figure 10A–F). Similarly, the frequency of OVA_257–264_-specific CD44^high^ CD8+ T cells in the spleen was increased by over four-fold in the group immunized with the combination of DMXAA + 522 relative to those in the control and other treatment groups (Figure 10G). In the lymph node, the frequency of OVA_257–264_-specific CD44^high^ CD8+ T cells nearly doubled with the combination treatment (Figure 10H). However, there was no change in the CD44^high^ CD8+ T cells in the tumor with any of the treatments (Figure 10I).

### 3.6. Natural Killer (NK) Cell Activation

**Figure 11:**
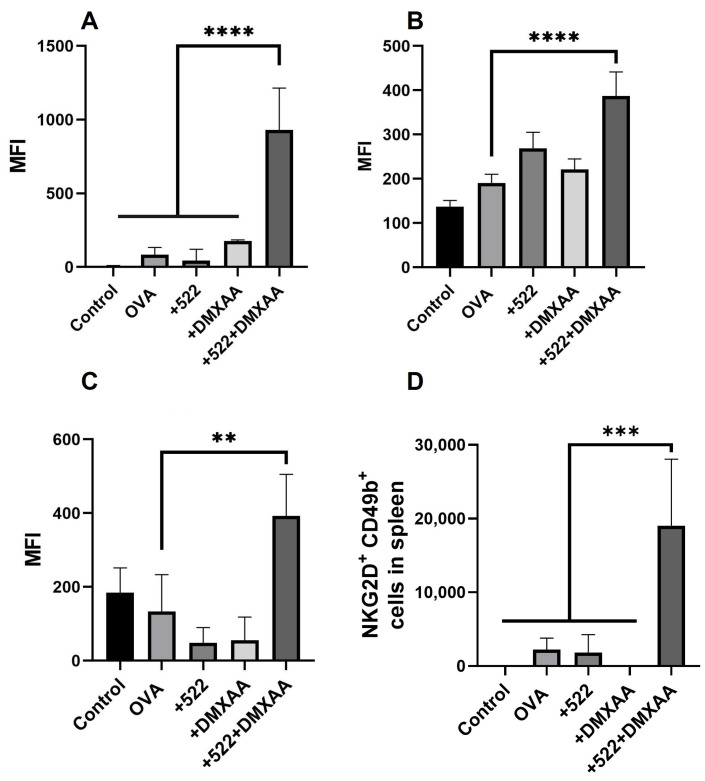
In vivo NK cell activation. NK cells were analyzed by flow cytometry. The figure shows the MFI of NKG2D^+^ NK cells in the spleen (**A**), CD69^+^ NK cells in spleen (**B**), frequency of NKG2D^+^ CD49b+ cells in spleen (**C**), and NKG2D^+^ NK cells in tumor (**D**). Results are reported as mean ± SD, *n* = 3, ** *p* < 0.01, *** *p* < 0.001, **** *p* < 0.0001 one-way ANOVA.

We further investigated the extent to which the combination of DMXAA and 522 activated NK cells by measuring the levels of the activation markers NKG2D and CD69 [24]. NKG2D expression significantly increased in the spleen (Figure 11A) compared to that in other groups. A significant increase in the activation marker CD69 was also observed in the spleen with the DMXAA + 522 treatments (Figure 11B). NKG2D expression was also significantly increased in the tumor for combination treatment compared to those in the control and individual treatment groups (Figure 11C). The levels of CD44 were not significantly different across the different treatment groups in any of the tissues (data not shown). Additionally, NKG2D or CD69 expression was not significantly different between the treatment groups in the lymph nodes. The frequency of CD49b^+^ NKG2D^+^ cells was significantly increased in the spleen (Figure 11D) in the DMXAA + 522 group, but similar trends were not seen across the lymph nodes or tumor.

## 4. Discussion

Immunomodulatory adjuvants such as TLR and STING agonists are keys component of cancer vaccines [25]. Due to central and peripheral tolerance mechanisms, tumor-associated antigens (TAA) are poorly immunogenic when administered alone and therefore elicit T cell anergy or regulatory T cell expansion [26]. By providing an appropriate ‘danger’ context, adjuvants serve to activate APCs that can then prime a vigorous TAA-specific adaptive immune response. Thus, adjuvants aid cancer vaccines by inducing, expanding, and maintaining a TAA-specific CD8 + T cell population [27,28]. Further, adjuvant formulations that elicit the expression of Th1-skewing cytokines such as IFN-γ, IL-12p70, and TNF-α enable more effective anticancer response [29]. Adjuvants can also substantially reduce the amount of antigen and/or frequency of immunizations required to achieve the desired immune responses [30,31].

We have previously reported a series of novel imidazoquinoline-based TLR7/8 agonists that are significantly more potent than both imiquimod and resiquimod in activating pro-inflammatory cytokine secretion [15,32]. We further demonstrated that the encapsulation of the TLR7/8-bispecific agonist 522 in polymeric nanoparticles resulted in the significant induction of both T cell and NK cell-mediated responses [14,21]. While free 522 was effective in activating DCs in vitro, only the nanoparticle-encapsulated form resulted in enhanced tumor growth inhibition in vivo. In the present study, we hypothesized that the immune stimulatory activity of 522 could be enhanced by combining it with a STING pathway agonist.

STING agonists can orchestrate anticancer immune response by APCs, which then prime and trigger Ag-specific T cell activation [33]. The mechanism through which STING activates APCs is not fully understood. It is thought that STING agonists mimic tumor-derived DNA, which can bind to cGAS in the cytoplasm of APCs, initiating a Type 1 IFN response [34,35]. IFNs act as a T cell receptor (TCR) signal in T cell stimulation.

A previous report investigated the combination of a TLR9 agonist [CpG oligodeoxynucleotide (CpG)] and a STING ligand [cyclic GMP-AMP (cGAMP)] based on the fact that CpG induces a weak IFN response while STING ligands induce type-2 immune responses [36]. The study showed that the combination of TLR9 and STING agonists resulted in an effective type-1 immune response, as demonstrated by improved antigen-specific IgG2c and IFN-γ production as well as cytotoxic CD8+ T cell responses. However, another study showed that the pre-stimulation of the cGAS-STING pathway in human plasmacytoid DCs reduced TLR9-mediated IFN production [37].

In the present study, we used DMXAA as a model STING agonist. DMXAA was originally developed as a vascular-disrupting agent and performed well as a STING agonist in preclinical studies [38]. However, it failed to show efficacy in clinical trials because it was found to be specific to mouse STING and does not activate human STING [17,39]. Although DMXAA failed in clinical trials, it has been utilized extensively in preclinical studies and is a good model compound (easy availability, reasonable cost, and more ‘drug-like’ properties compared to cyclic oligonucleotides) for pre-clinical studies [40].

In vitro BMDC assays clearly demonstrated the enhanced activation of DCs with the combination therapy based on both co-stimulatory molecule expression and pro-inflammatory cytokine secretion. It was interesting to note that the combination treatment resulted in substantially elevated levels of IL-12, a key cytokine that orchestrates the Th1 anticancer immune response [41]. IL-12 stimulates the growth and cytotoxicity of activated NK cells and CD8^+^ T cells and enhances the production of IFN-γ from these cells. IL-12 also stimulates the differentiation of naïve CD4^+^ cells to the Th1 phenotype. Both IL-6 and TNF-α, which have dual roles in immune response to tumor, were also elevated with the combination treatment. TNF-α can have either anti-cancer activity or behave as an immunosuppressive cytokine [42]**.** Recent studies show that TNF can facilitate the accumulation of regulatory T lymphocytes (Tregs) as well as myeloid-derived suppressor cells (MDSC) [43,44]. Similarly, IL-6 can support tumor proliferation and metastatic dissemination [45,46,47]. However, more recent studies show IL-6 signaling can inhibit tumor growth by enhancing the tumor and lymph node trafficking of cytotoxic T cells [48,49].

Similar to that seen in our previous studies, free 522 was not effective in inhibiting tumor growth in vivo. Similarly, free DMXAA did not affect tumor growth (in the B16F10 model) or only inhibited the growth marginally (MB49 model). In previous studies, DMXAA by itself demonstrated some tumor growth inhibition in the B16F10 model [50,51]. This could be because of either the higher doses used in those studies or because DMXAA was administered directly into STING-positive tumors. For example, the administration of a single 500 μg dose (equivalent to ~25 mg/kg) of DMXAA intratumorally in B16 tumors expressing a model antigen SIYRYYGL resulted in a potent antitumor effect. Similarly, a single intraperitoneal dose (25 mg/kg) of DMXAA [52] or two intravenous doses [53] resulted in the modest inhibition of tumor growth.

The enhanced anticancer therapeutic effect of the DMXAA + 522 combination could be mediated, at least partly, through the activation of antigen-specific immune response. The use of OVA as the model antigen allowed us to monitor OVA-specific responses both systemically (in the spleen) and locally (in lymph nodes and tumor). We observed the presence of mature (CD86^+^) and activated (CD40^+^) DCs cross-presenting OVA-derived peptide SIINFEKL via MHC I in the spleen. The DCs present in the tumor were positive for H-2K^b,^ but not for CD40 or CD86, while the DCs in lymph nodes were positive for CD40. Cross-presentation via MHC I is a critical requirement for the priming and activation of antigen-specific CD8+ T cells [54]. These studies suggest that the priming and activation of OVA-specific CD8+ T cells may occur in the spleen rather than in the draining lymph nodes or within the TME. It is also possible that activated DCs cross-presenting OVA peptide migrated away from the lymph node to the spleen by day 8. This is corroborated by the presence of OVA-specific activated and memory CD8+ T cells in the spleen, in addition to lymph nodes.

We note here that it is not possible to fully attribute the observed therapeutic benefits to antigen-specific immune response, because our studies did not include adjuvant-only control groups. Both TLR7/8 and STING agonists have other immunostimulatory activities that are independent of their ability to activate antigen-presenting cells [55,56]. For example, TLR7/8 agonists can directly inhibit IL17 signaling in Th17 cells which can contribute directly to tumor growth inhibition [57]. Similarly, the STING pathway can modulate the tumor vasculature and augment adaptive immunity by supporting tertiary lymphoid structure development [58]. Additional studies with adjuvant-only groups are needed to further evaluate these effects. DMXAA + 522 treatment resulted in other anticancer immunostimulatory effects, including increased numbers of activated NK cells in the spleen and tumor. We previously reported the enhanced activation of NK cells with our TLR7/8 agonists [59]. In that study, we observed the significantly higher induction of cytokines, considered key drivers of NK cell activation including IFN-α and IFN-β (activate NK cells), as well as IL-2, IL-15 (promote NK cell survival, proliferation, and activation), and IL-12 (promote optimal cytokine production by NK cells). Interestingly, DMXAA appears to not have any NK cell activity, which is in agreement with a previous study showing that the therapeutic effect of DMXAA was lost in mice depleted of CD8^+^ T cells but not in those depleted of NK cells.

The improved antitumor response with the 522 + DMXAA adjuvant combination can be correlated to the enhanced systemic IFNγ levels achieved with this combination. Many cancer immunotherapies, including checkpoint inhibitors, induce IFNγ production by various immune cells, especially activated T cells and NK cells [60]. Other studies have shown that defects in IFNγ signaling result in resistance to immunotherapy [61]. Thus, IFNγ is likely a key mediator of efficacy for cancer immunotherapy. However, IFNγ can also have potentially toxic effects on antitumor immune cells. Thus, IFNγ may have both immunostimulatory, anti-tumor effects or immunosuppressive, pro-tumor effects, depending on the context in which it is produced [62]. The direction of this anti- vs. pro-tumorigenic response may depend on the duration and magnitude of IFNγ production/signaling. Acute IFNγ exposure likely results in the activation of an anti-tumor immune response (the recruitment and activation of antigen-presenting cells, T cell priming and activation, NK cell activation, and tumor cell killing). However, prolonged IFNγ exposure can promote pro-tumorigenic effects (checkpoint activation, angiogenesis, and tumor cell proliferation) [63]. Thus, it is important to monitor the duration and extent of IFNγ production following any immunotherapy to bias the response towards anti-tumorigenic effects.

We also observed a reduction in the number of pro-tumorigenic M2 phenotypic (CD206^+^) macrophages and an increase in tumoricidal M1 phenotypic (CD80^+^) macrophages in the spleen, lymph node, and tumor with the combination treatment. Other studies show TLR7/8 agonists such as R848 can effectively repolarize M2 macrophages into the M1 phenotype [64]. However, in our studies, DMXAA appears to drive the macrophage polarization since free 522 did not have any effect on the macrophage phenotype. Previous studies show that DMXAA can effectively reprogram macrophages from the M2 to the M1 phenotype and augment the efficacy of immunotherapy against established tumors [65,66].

## 5. Conclusion and Future Studies

Our studies show that the combination of DMXAA and 522 is effective in activating both innate and adaptive immune responses. While the current studies demonstrate the potential of TLR7/8 and STING agonist combination as vaccine adjuvants, we did not observe complete tumor eradication in any of the treated mice. This suggests that further improvements in efficacy are warranted and possible. Our current studies examined the subcutaneous delivery of the two agonists, with the goal of targeting peripheral DCs. Most previous studies utilized the intratumoral administration of STING agonists, which are also known to directly induce apoptosis in tumor cells. Thus, it is important to examine the effect of the route of administration for the two agonists. It is also possible the two molecules have varying drug disposition profiles, which could limit their ability to act in a synergistic or additive fashion. Future studies will examine the pharmacokinetics and tissue levels of the two agents following different routes of dosing. As demonstrated in our previous reports, the encapsulation of the two agonists in polymeric nanoparticles could further improve their effectiveness. The co-encapsulation of the two agents in the same formulation would also allow for their simultaneous delivery to target tissues.

## Data Availability

The data that support the findings of this study will be made available by the corresponding author upon reasonable request.

## Supplementary Materials

The following supporting information can be downloaded at: https://www.mdpi.com/article/10.3390/cancers14246091/s1, Figure S1: List of markers used for flow cytometry studies and gating strategies for different cell populations; Figure S2: BMDC costimulatory molecule expression and cytokine secretion; Figure S3: Different cytokines measured in the blood serum on day 7 after five consecutive doses day 1 through day 5.
