# Supplementary material for: Combination of STING and TLR 7/8 Agonists as Vaccine Adjuvants for Cancer Immunotherapy
Source: Cancers (Basel). 2022 Dec 11;14(24):6091. doi: 10.3390/cancers14246091 (PMC9777055; doi:10.3390/cancers14246091)
Supplement: Supplementary file 1 [file cancers-14-06091-s001.zip › cancers-2063046-supplementary.pdf]

# Supplementary information

## Combination of STING and TLR 7/8 agonists as vaccine adjuvants for cancer immunotherapy

Shubhmita Bhatnagar[1], Vishnu Revuri[1], Manan Shah[1,2], Peter Larson[3], Zekun Shao[1,2], Daohai Yu[4], Swayam Prabha[5,6], Thomas S. Griffith[7], David Ferguson[3], Jayanth Panyam[1,2,6]*

[1]Department of Pharmaceutical Sciences, School of Pharmacy, Temple University, Philadelphia PA 19140
[2]Department of Pharmaceutics, College of Pharmacy, University of Minnesota, Minneapolis MN 55455
[3]Department of Medicinal Chemistry, College of Pharmacy, University of Minnesota, Minneapolis MN 55455
[4]Center for Biostatistics and Epidemiology, Lewis-Katz School of Medicine, Temple University, Philadelphia PA 19140
[5]Fels Cancer Institute for Personalized Medicine, Lewis-Katz School of Medicine, Temple University, Philadelphia PA 19140
[6]Fox Chase Comprehensive Cancer Institute, Temple University, Philadelphia PA 19111
[7]Department of Urology, Medical School, University of Minnesota, Minneapolis MN 55455

| Marker | Immune Cell Subsets | Function |
|---|---|---|
| CD45 | All immune cells | Leukocyte marker |
| CD3 | T cells | Lineage Marker |
| CD49 | NK cells | Lineage marker |
| CD4 | CD4 T cells | Lineage marker |
| CD8 | CD8 T cells | Lineage Marker |
| PD-1 | T cells | Checkpoint molecule |
| CD69 | T, NK | Activation marker |
| CD44 | T cells | Memory & Activation |
| Tetramer | CD8 T cells | OVA-specific CTLs |
| NKG2D | NK cells | Activation marker |
| CD11c | DCs – high,<br>Mφ, B cells , Monocytes – low | Lineage marker |
| F4/80 | Mφ | Lineage Marker |
| CD19 | B cells | Lineage marker |
| H-2K$^b$ SIINFEKL | DCs, Mφ, B cells | Binds to OVA on MHC I expressed by APCs |
| PD-L1 | APCs and tumor cells | Checkpoint molecule |
| CD40 | DCs, Mφ, B cells | Activation marker |
| CD80 | DCs, Mφ, B cells | Activation, M1 marker (F4/80+ CD206- CD80+) |
| CD86 | DCs, Mφ, B cells | Activation marker |
| CD206 | Immunosuppressive Mφ | M2 marker |

| Cell Subset | Markers |
|---|---|
| DCs | CD45+ F4/80- CD11c+ |
| B cells | CD45+ F4/80 – CD19+ |
| F4/80 | CD45+ F4/80+ CD11c- |
| M2 macrophages | CD45+ F4/80+ CD80- CD206+ |
| CD4 T cells | CD45+ CD3+ CD4+ CD8- |
| CD8 T cells | CD45+ CD3+ CD4- CD8+ |
| NK Cells | CD45+ CD3- CD49b+ NKG2D+ |
| Tumor cells | CD45- |

**Figure S1**. List of markers used for flow cytometry studies and gating strategies for different cell populations.

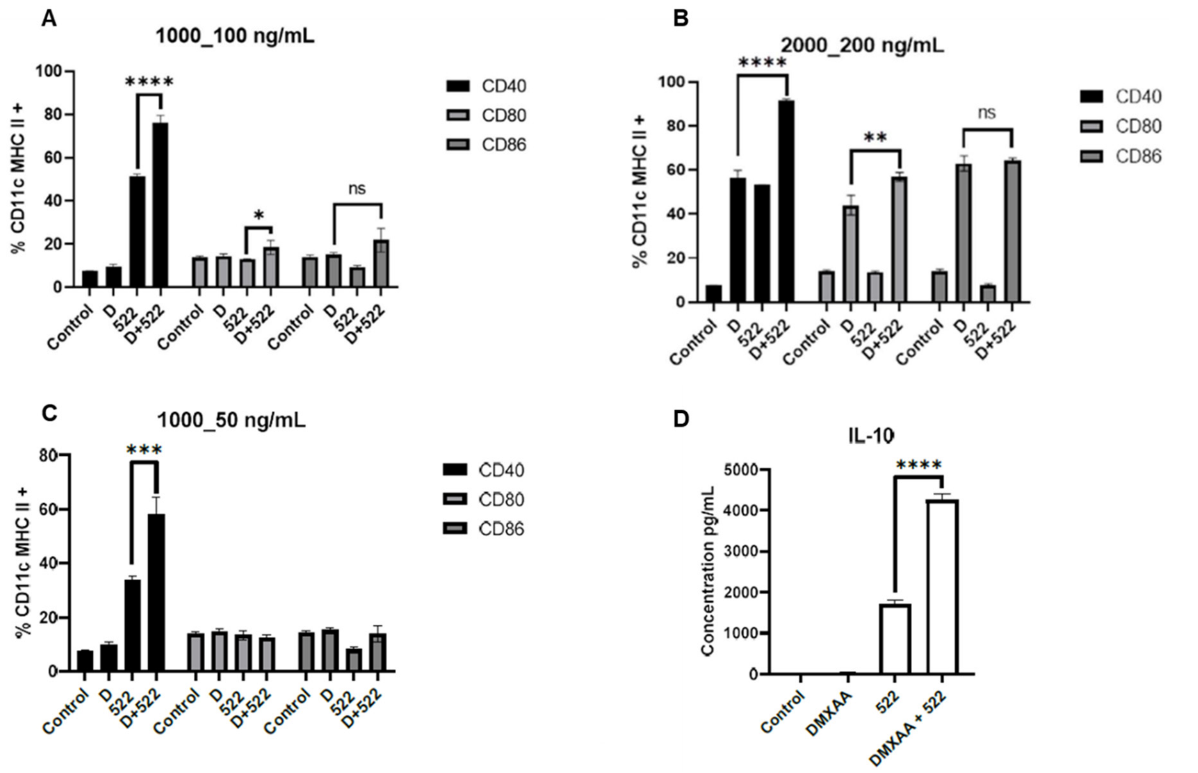


**Figure S2.** BMDC costimulatory molecule expression and cytokine secretion. BMDCs were incubated with DMXAA (1000 ng/ml, 2000 ng/ml), 522 (50 ng/ml, 100 ng/ml , 200 ng/ml) and DMXAA + 522 (1000_100, 1000_50, 2000_200) for 24 h and costimulatory molecules CD40, CD80 and CD86 were measured by flow cytometry (A-C). CD11c and MHCII double positive cells identified as BMDCs in the population and were gated further for analysis. A population was selected and gated on the MHCII vs CD11c axis. Double positive events were analyzed for CD86, CD80 and CD40 expression. BMDCs were incubated with DMXAA (2000 ng/ml), 522 (50 ng/ml) and DMXAA + 522 (2000_50 ng/ml) for 24 h and IL-10 were measured by ELISA (D). Results are reported as mean ± SD, n=3, ****p<0.0001, ***p<0.001, One-way ANOVA.

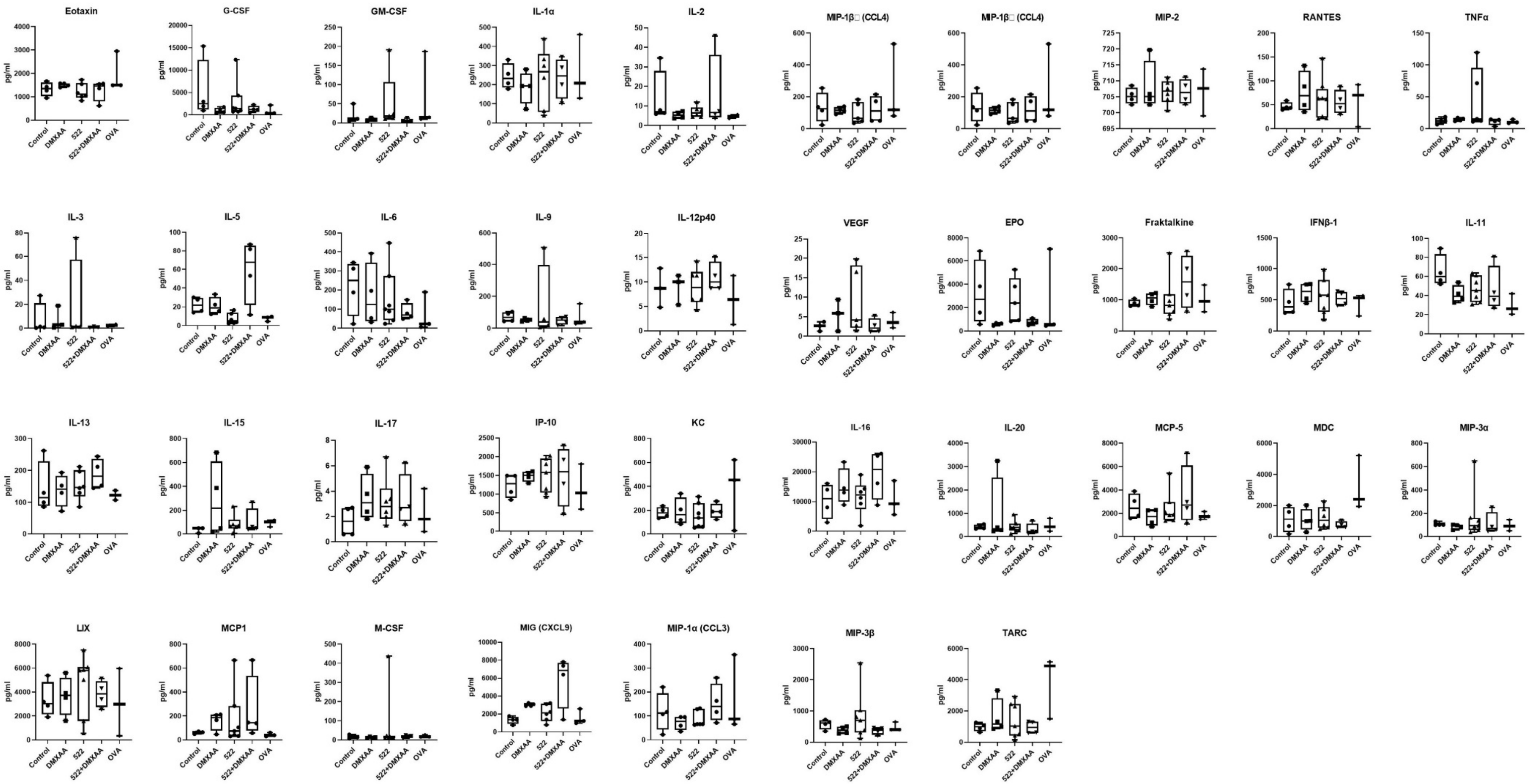


Figure S3. Different cytokines measured in the blood serum on day 7 after five consecutive doses day 1 through day 5.
